# COVID‐19 and acute pancreatitis: A systematic review

**DOI:** 10.1002/jgh3.12729

**Published:** 2022-04-14

**Authors:** Oyedotun Ikechukwu Babajide, Ekwevugbe Ochuko Ogbon, Anuoluwapo Adelodun, Olufunso Agbalajobi, Yetunde Ogunsesan

**Affiliations:** ^1^ Department of Internal Medicine Interfaith Medical Center, One Brooklyn Health System Brooklyn New York USA; ^2^ Department of Internal Medicine Michigan State University, Hurley Medical Center Flint Michigan USA; ^3^ Department of Internal Medicine Harlem Hospital Center, Columbia University New York City New York USA; ^4^ Department of Medicine / Division of Internal Medicine University of Pittsburgh Medical Center Pittsburgh Pennsylvania USA; ^5^ Winthrop Rockefeller Cancer Institute University of Arkansas for Medical Sciences (UAMS) Myeloma Center Little Rock Arkansas USA

**Keywords:** abdominal pain, acute pancreatitis, coronavirus disease 2019, pneumonia, severe acute respiratory syndrome coronavirus 2

## Abstract

We aimed to systematically review the relationship between severe acute respiratory syndrome coronavirus 2 (SARS‐CoV‐2) infection and acute pancreatitis (AP). The global pandemic of coronavirus disease 2019 (COVID‐19) caused by SARS‐CoV‐2 infection causes respiratory symptoms and notably also affects the gastrointestinal (GI) system. A systematic review of the available literature on the topic was performed with a search key using the terms “SARS COV 2,” “Pancreatitis,” “COVID‐19” and synonyms. The search was conducted on 27 December 2020 using PubMed, EMBASE, CENTRAL, Web of Science, and Scopus. A meta‐analysis was not conducted due to the low quality and poor comparability of the studies. We reviewed 66 studies that reported data on patients with polymerase chain reaction‐confirmed SARS‐CoV‐2 infection and AP using the Atlanta Criteria. Our evaluation revealed a wide age range and diverse clinical presentation of COVID‐19 with or without symptoms of AP, some of which preceded typical COVID‐19 symptoms. We observed a myriad of complications and one study revealed that patients with both conditions were more likely to require mechanical ventilation and had longer lengths of hospital stay compared with patients with AP without COVID‐19. Treatment for AP was mostly supportive, with varied therapies employed for COVID‐19. Most cases were considered idiopathic and presumed to be SARS‐CoV‐2‐induced as established etiological factors were not reported. AP should be considered in COVID‐19 patients, especially in those exhibiting GI symptoms. Evidence to establish a causal relationship between SARS‐CoV‐2 infection and AP is currently lacking.

## Graphical abstract

We aimed to systematically review the relationship between severe acute respiratory syndrome coronavirus 2 (SARS‐CoV‐2) infection and acute pancreatitis, seeing that the coronavirus disease 2019 (COVID‐19) caused by SARS‐CoV‐2 infection also affects the gastrointestinal (GI) system. Acute pancreatitis should be considered in COVID‐19 patients, especially in those exhibiting GI symptoms; however, evidence to establish a causal relationship between SARS‐CoV‐2 infection and AP is currently lacking.

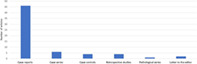

## Introduction

In December 2019, coronavirus disease 2019 (COVID‐19) caused by severe acute respiratory syndrome coronavirus 2 (SARS‐CoV‐2) was first described, and subsequently became a global pandemic. As of July 13, 2021, over 187 million cases had been confirmed globally and were responsible for more than 4 million deaths worldwide.^1^ The typical presentation of symptomatic COVID‐19 includes fever, cough, myalgia, and headache, with other features such as dyspnea, sore throat, diarrhea, nausea, and vomiting. Abnormalities in smell (anosmia) and taste (dysgeusia) were also identified.^2^, ^3^ Specifically, gastrointestinal (GI) involvement of COVID‐19 has been recognized,^4^, ^5^, ^6^, ^7^ and the viral RNA has repeatedly been detected in the stool samples of infected individuals.^8^, ^9^ In a meta‐analysis^10^ of 6686 patients with COVID‐19, the pooled prevalence of digestive symptoms was 15%, with nausea or vomiting, diarrhea, and loss of appetite being the three most common symptoms. Exactly 10% of patients presented with GI symptoms alone without respiratory features.^10^ These GI involvements are thought to be mediated by the expression of angiotensin‐converting enzyme 2 (ACE‐2) on the GI tract,^11^, ^12^, ^13^ which is the viral receptor for the entry of SARS‐CoV‐2.^14^, ^15^ Attention to pancreatic injury has been lacking until recently. COVID‐19 associated pancreatic injury has been suggested, but its correlation with pancreatic disease is unclear. The expression of ACE‐2 in the pancreas^13^, ^16^ in both exocrine glands and islets renders the pancreas a potential target for SARS‐CoV‐2, the RNA of which has also been detected in pancreatic pseudocyst fluid samples, according to one case report.^17^ Several case reports and retrospective studies of pancreatic injury and acute pancreatitis (AP) caused by the novel coronavirus have been reported. The clinical findings of acute pancreatic injury in these studies raise the question of whether the virus has a tropism for pancreatic tissue and whether it plays a role in the incidence of pancreatic disease. This systematic review aims to explore the relationship between SARS‐CoV‐2 infection, (COVID‐19) and AP.

## Methods

We performed a systematic review of the available literature on the topic with a search key using the terms “SARS COV 2,” “Pancreatitis,” “COVID‐19” and synonyms. The systematic search was conducted on 27 December 2020 using PubMed, EMBASE, CENTRAL, Web of Science, and Scopus. Due to the low quality and poor comparability of the studies, a meta‐analysis was not conducted.

### Eligibility criteria

All case reports and case series, regardless of number, were considered eligible if they contained the original data on at least one SARS‐CoV‐2‐infected individual diagnosed with AP. COVID infection was diagnosed with polymerase chain reaction (PCR), while AP was diagnosed using the revised Atlanta Classification for Acute Pancreatitis. Only human studies were included. Studies containing solely animal or in vitro data were excluded. Systematic reviews and meta‐analyses were excluded due to duplication of data.

### Systematic search and selection; data extraction

The systematic search was conducted in five databases: EMBASE, MEDLINE (via PubMed), CENTRAL, Web of Science, and Scopus. The systematic search was conducted on 27 December 2020. The search was restricted to 2020, and no other filters were applied. Citations were exported to a reference management program (EndNote X9, Clarivate Analytics, Philadelphia, Pennsylvania, USA). Two independent review authors (Ekwevugbe Ochuko Ogbon and Oyedotun Ikechukwu Babajide) used rayyan to double blind and conducted the selection by title, abstract, and full text based on the previously disclosed, predetermined set of rules. The articles were subsequently unblinded and conflicts were resolved by the third and fourth reviewers (Anuoluwapo Adelodun and Yetunde Ogunsesan) who decided whether to include or exclude these articles.

Citing articles and references in the studies assessed for eligibility in the full‐text phase were reviewed to identify any additional eligible records. Data were extracted from all eligible studies into a standardized Excel sheet designed on the basis of recommendations from the Cochrane Collaboration.

## Results

**Figure 1:**
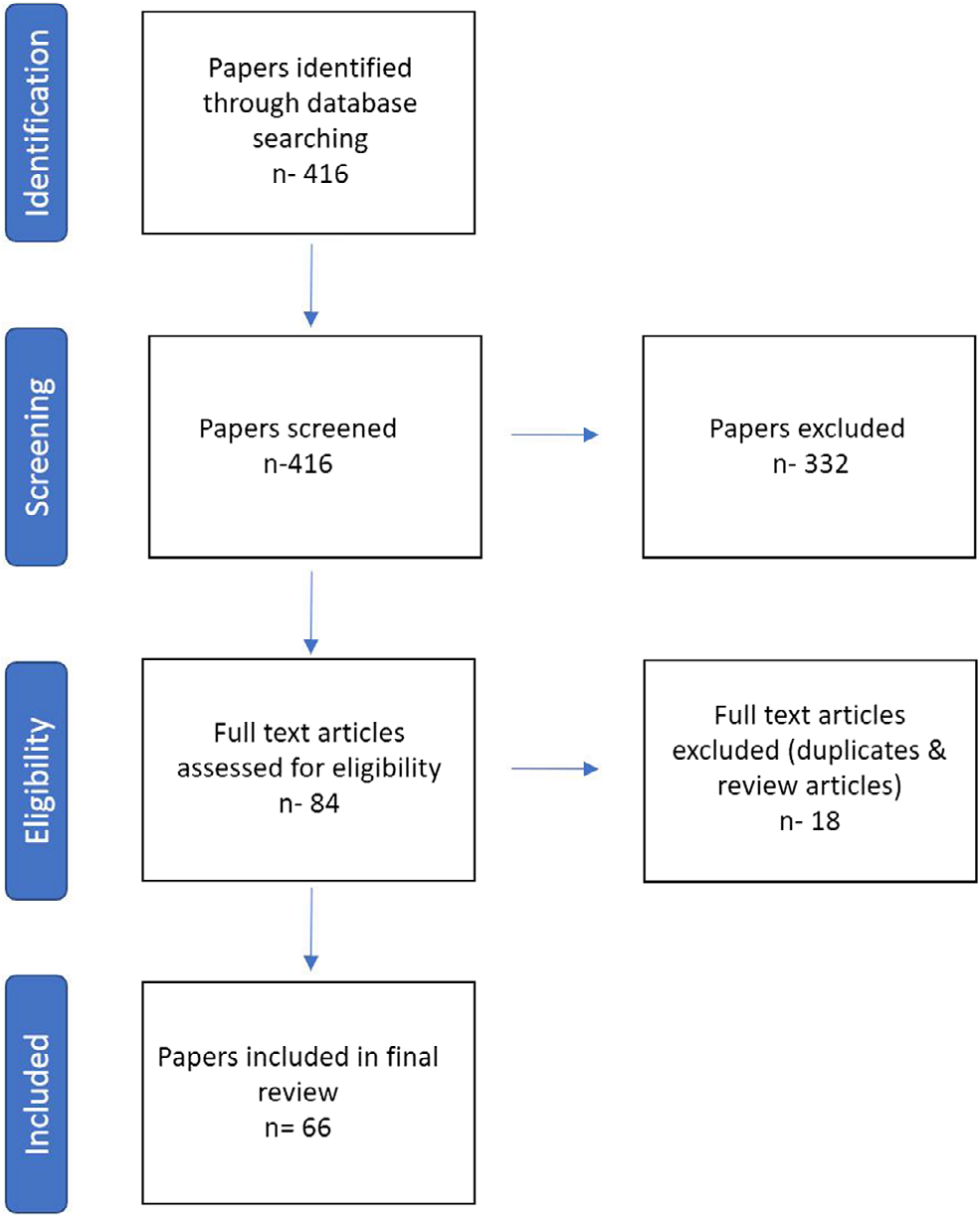
Flowchart: Data collection and selection of studies

**Figure 2:**
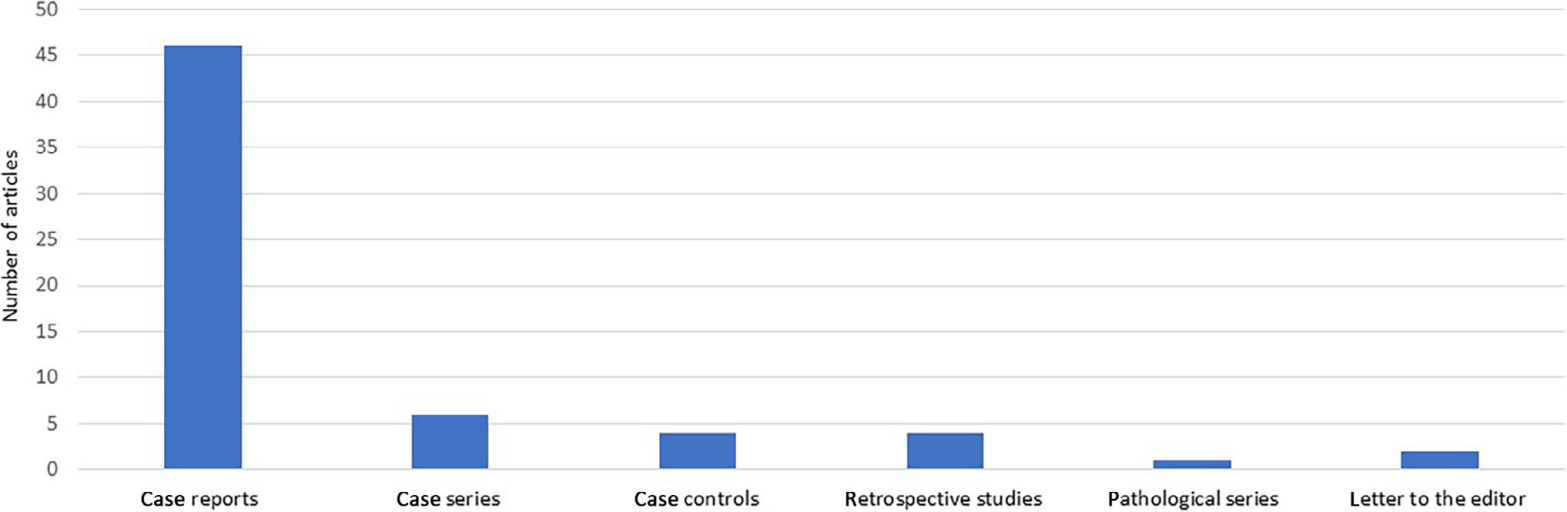
Types of studies in the review. (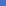), Study types.

**Figure 3:**
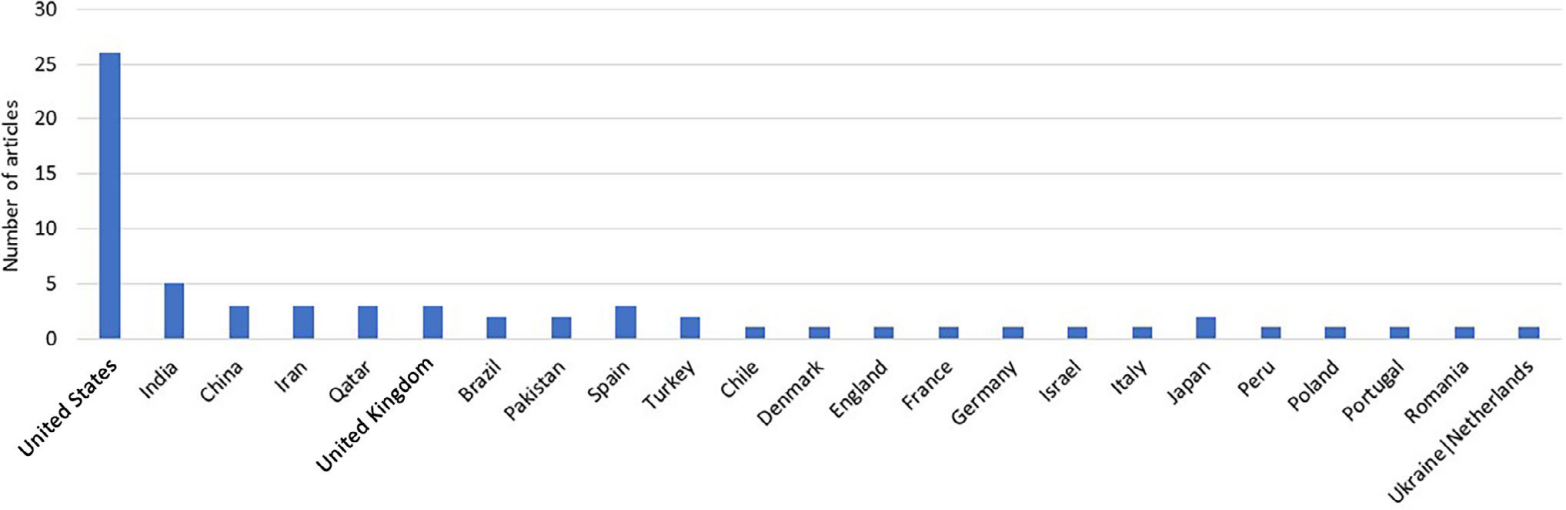
Country of origin of articles. (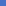), Country of origin.

The initial search yielded 416 articles. After excluding duplicates and review articles, 66 studies—Figure 1 (49 case reports, 6 case series, 4 retrospective studies, 4 case controls, 2 Letters to editor, 1 Pathologic series—Fig. 2) were included in the pooled analysis. Twenty‐six of these studies were from the United States and five from India, while all the other studies were from other countries as listed in Figure 3. A myriad of different comorbidities was seen, as shown in Table 1.

**Table 1:** Distribution of comorbidities

Comorbidity	Count
	28
Asthma	1
BPH	1
CHF	1
Cholecystectomy	1
CKD	2
COPD	1
Diabetes mellitus	6
Dyslipidemia	1
Hypertension	11
Hypothyroidism	1
Ischemic heart disease	1
Obesity	3
Osteoporosis	1
Post HELLP syndrome	1
Previous pancreatitis	1
Prior cholecystectomy	3
Thrombophilia	1
Grand total	65

BPH, benign prostatic hypertrophy; CHF, congestive heart failure; CKD, chronic kidney disease; COPD, chronic obstructive pulmonary disease; HELLP, hemolysis, elevated liver enzymes, and low platelets.

There were a total of 151 086 COVID positive patients and 256 (0.16%) were diagnosed with pancreatitis based on clinical and radiologic findings. Out of the studies with the following data, we had more male (110, 44%) than female patients (65, 25.3%) while the gender of 81 patients were unspecified. The modal age was 53, with a median age of 50. The youngest case was a 5‐year‐old male and the oldest a 97‐year‐old male. The average length of stay was 14.5 days, with LOS ranging from 3 to 64 days across 32 studies. Of these patients, 45 were admitted to the intensive care unit (ICU). A total of 143 recovered, 25 died, and the status of 88 patients remained unknown due to data not being available.

Clinical presentations were mostly diverse with—(fever, malaise, shortness of breath, and cough) and/or with symptoms of AP—abdominal pain and vomiting. In some cases, symptoms of pancreatitis preceded that of COVID‐19 infection.^18^

Imaging studies showed mostly bilateral opacities on chest imaging and a swollen pancreas with peripancreatic inflammatory changes on abdominal computed tomography (CT) scans. Laboratory studies showed elevated lipase and/or amylase among other derangements like elevated liver enzymes, and in one case, laboratory findings suggest DKA.^19^

Treatment modalities for AP were focused mostly on supportive care with intravenous hydration and analgesia; however, some cases required surgery for complications of AP.^20^ Management of COVID‐19 on the other hand included different therapies like steroids, antibiotics, antivirals, antiparasitics, and in some instances, monoclonal antibodies.

Complications varied from necrotizing pancreatitis, diffuse hemorrhagic pancreatitis, ARDS, multiorgan failure, and in one study preterm delivery.

One study also found SARS‐CoV2 RNA detection in a pancreatic pseudocyst sample.^17^

## Discussion

The COVID‐19 virus has been a major part of our lives for the better part of 2020 and 2021. Its effects are far reaching in time and space as evidenced by the fact that it was discovered in the Wuhan province of China in 2019 but is still a global pandemic in 2021. Our review revealed global consistency as we found cases of AP in COVID‐19 infection published around the world with reports from 22 countries reviewed.

Like its global spread, this virus has been shown to have widespread effects on the human body. Initially thought to affect only the respiratory system, reports of multiple organ involvement, especially organs that express the ACE‐2 cell receptor, are now commonplace. Therefore, expression of the ACE‐2 receptor may have an important role in understanding the presentation and progression of this disease. ACE‐2 receptors are expressed in the GI epithelial cells and COVID‐19 virus elements have been isolated in stool samples of infected patients.^8^, ^9^

One of several organs that express the ACE‐2 receptor is the pancreas, with receptors being found in the ductal as well as the acinar and islet cells.

In one study, SARS‐CoV2 RNA was detected in a pancreatic pseudocyst sample^17^ and raised the question of whether there is any tropism and if one can conclude that AP during a COVID‐19 infection could be a form of viral pancreatitis.

The most common cause of AP is gallstones^21^; other common causes are alcohol and hypertriglyceridemia, especially with triglycerides greater than 1000 mg/dL.

Rare causes of AP include medications, biliary obstruction, hypercalcemia, and infections. Viruses including coxsackie, mumps, and measles viruses have been implicated as causes of AP^22^ and the SARS‐CoV‐2 has also been isolated from pancreatic samples during autopsies of infected patients^23^; however, there has been no direct evidence that COVID‐19 infection causes pancreatitis. Some studies have shown significant pancreatic injury as evidenced by elevations of serum amylase/lipase^24^ and in another study about one‐third of patients with COVID‐19 had pancreatic injury.^25^

Although pancreatic injury in patients with COVID‐19 is common, actual AP is not much so. In a retrospective cohort study of hospitalized patients with COVID‐19, where over 10 000 cases were looked at, AP had a point prevalence of 0.27%. The etiology of pancreatitis in the COVID‐19 group was mostly classified as being idiopathic as compared with other causes such as gallstones and alcohol use. The same study also showed a higher proportion of pancreatitis in the Black and Hispanic patients hospitalized with COVID‐19.^26^

In our review, one study revealed that patients with AP who were also found to be positive for COVID‐19 were more likely to require mechanical ventilation and had longer lengths of hospital stay compared with patients with AP without COVID‐19 (odds ratio [OR], 5.65; *P* = 0.01 and OR, 3.22; *P* = 0.009, respectively).^26^

There were also a myriad of complications seen in patients with COVID‐19 infection and AP such as necrotizing pancreatitis, diffuse hemorrhagic pancreatitis, ARDS, multiorgan failure, and in one study preterm delivery.^27^ However, we did not find enough data to conclude that patients with COVID‐19 were more likely to have complications associated with AP as compared with patients with AP alone.

In most of the cases reviewed, patients were not reported as having the usual risk factors associated with AP like alcoholism, hypertriglyceridemia, and gallstones, but it is unclear if proper evaluation was done to rule out other causes of AP as most of the cases were labeled as idiopathic.

Another limitation encountered was the lack of standard therapies for treatment of COVID‐19 pneumonia. Various therapies ranging from steroids, antibiotics, antivirals, and in some instances monoclonal antibodies, howbeit rarely have been implicated as having AP as an adverse effect, and this can be a limitation in identifying probable associations between COVID infection and AP.

In conclusion, in the light of the first two waves and possibly a third wave of this pandemic, more research is needed to study the relationship between COVID‐19 infection and AP. There is currently no evidence to point to an association but that can be blamed on the paucity of data and also AP being infrequent in patients with COVID‐19 infection. There is a need for more robust studies to find out the incidence of AP in COVID‐19 infection. These studies may lead to answering some pertinent questions such as the possibility of an association between the two diseases and the effect of such association on increased morbidity and mortality.

## Data Availability

A summary of included studies/papers can be obtained by sending an email to the corresponding author.
